# Prediction of large‐for‐gestational‐age infant by fetal growth charts and hemoglobin A1c level in pregnancy complicated by pregestational diabetes

**DOI:** 10.1002/uog.26071

**Published:** 2022-12-01

**Authors:** M. K. Kiefer, M. M. Finneran, C. A. Ware, P. Foy, S. F. Thung, S. G. Gabbe, M. B. Landon, W. A. Grobman, K. K. Venkatesh

**Affiliations:** ^1^ Department of Obstetrics and Gynecology, Division of Maternal–Fetal Medicine The Ohio State University Columbus OH USA; ^2^ Department of Obstetrics and Gynecology, Division of Maternal–Fetal Medicine Medical University of South Carolina Charleston SC USA

**Keywords:** diabetes, diabetes mellitus, glycosylated hemoglobin, hemoglobin A1c, large‐for‐gestational age, LGA, macrosomia, pregestational diabetes, pregnancy

## Abstract

**Objectives:**

To compare the ability of three fetal growth charts (Fetal Medicine Foundation (FMF), Hadlock and National Institutes of Child Health and Human Development (NICHD) race/ethnicity‐specific) to predict large‐for‐gestational age (LGA) at birth in pregnant individuals with pregestational diabetes, and to determine whether inclusion of hemoglobin A1c (HbA1c) level improves the predictive performance of the growth charts.

**Methods:**

This was a retrospective analysis of individuals with Type‐1 or Type‐2 diabetes with a singleton pregnancy that resulted in a non‐anomalous live birth. Fetal biometry was performed between 28 + 0 and 36 + 6 weeks of gestation. The primary exposure was suspected LGA, defined as estimated fetal weight ≥ 90^th^ percentile using the Hadlock (Formula C), FMF and NICHD growth charts. The primary outcome was LGA at birth, defined as birth weight ≥ 90^th^ percentile, using 2017 USA natality reference data. The performance of the three growth charts to predict LGA at birth, alone and in combination with HbA1c as a continuous measure, was assessed using the area under the receiver‐operating‐characteristics curve (AUC), sensitivity, specificity, positive predictive value and negative predictive value.

**Results:**

Of 358 assessed pregnant individuals with pregestational diabetes (34% with Type 1 and 66% with Type 2), 147 (41%) had a LGA infant at birth. Suspected LGA was identified in 123 (34.4%) by the Hadlock, 152 (42.5%) by the FMF and 152 (42.5%) by the NICHD growth chart. The FMF growth chart had the highest sensitivity (77% *vs* 69% (NICHD) *vs* 63% (Hadlock)) and the Hadlock growth chart had the highest specificity (86% *vs* 76% (NICHD) and 82% (FMF)) for predicting LGA at birth. The FMF growth chart had a significantly higher AUC (0.79 (95% CI, 0.74–0.84)) for LGA at birth compared with the NICHD (AUC, 0.72 (95% CI, 0.68–0.77); *P* < 0.001) and Hadlock (AUC, 0.75 (95% CI, 0.70–0.79); *P* < 0.01) growth charts. Prediction of LGA improved for all three growth charts with the inclusion of HbA1c measurement in comparison to each growth chart alone (*P* < 0.001 for all); the FMF growth chart remained more predictive of LGA at birth (AUC, 0.85 (95% CI, 0.81–0.90)) compared with the NICHD (AUC, 0.79 (95% CI, 0.73–0.84)) and Hadlock (AUC, 0.81 (95% CI, 0.76–0.86)) growth charts.

**Conclusions:**

The FMF fetal growth chart had the best predictive performance for LGA at birth in comparison with the Hadlock and NICHD race/ethnicity‐specific growth charts in pregnant individuals with pregestational diabetes. Inclusion of HbA1c improved further the prediction of LGA for all three charts. © 2022 The Authors. Ultrasound in Obstetrics & Gynecology published by John Wiley & Sons Ltd on behalf of International Society of Ultrasound in Obstetrics and Gynecology.

**CONTRIBUTION**

**What are the novel findings of this work?**

In pregnant individuals with pregestational diabetes, the Fetal Medicine Foundation (FMF) fetal growth chart had the best predictive performance for large‐for‐gestational age (LGA) at birth compared with the Hadlock and National Institutes of Child Health and Human Development race/ethnicity‐specific growth charts. Prediction of LGA improved further with the measurement of hemoglobin A1c (HbA1c) in addition to fetal biometry.

**What are the clinical implications of this work?**

Improving the prediction of LGA using the most accurate prenatal fetal growth chart (such as the FMF chart) in combination with HbA1c could assist with clinical decision‐making with regard to the prenatal management of macrosomia in pregnancies complicated by pregestational diabetes. Whether improved prediction results in improved clinical outcome, including consequent glycemic control and associated perinatal outcome, requires further study.

## INTRODUCTION

More than one in three infants born to pregnant individuals with pregestational diabetes are diagnosed as large‐for‐gestational age (LGA) at birth^1^. Pregnant individuals with LGA are at increased risk for fetal shoulder dystocia, postpartum hemorrhage, Cesarean delivery and obstetric anal sphincter injuries^2^, ^3^, ^4^. LGA infants are at increased risk for neonatal intensive care unit admission, low Apgar scores, brachial plexus injury, neonatal hypoglycemia, stillbirth and childhood obesity^5^, ^6^, ^7^. Accurate prediction of LGA infants is potentially useful to improve glycemic control and to identify individuals at increased risk for adverse outcome^2^.

Prenatal ultrasound can be used to identify infants at greater risk for LGA at birth^8^. Multiple validated fetal growth charts have been developed, including the Hadlock^9^, ^10^, National Institutes of Child Health and Human Development (NICHD) race/ethnicity‐specific^11^ and the Fetal Medicine Foundation (FMF)^12^ growth charts. The Hadlock growth chart was published in 1985 and was developed based primarily on a group of low‐risk, healthy and non‐Hispanic white individuals of higher socioeconomic status^9^, ^10^, ^13^. The NICHD race/ethnicity‐specific growth chart was published in 2015 and was designed to account for the variability of normative birth weights (BW) by self‐reported maternal race and ethnicity^11^, ^14^. The FMF growth chart was published in 2018 and was developed to improve the identification of fetal growth restriction^12^.

Prior studies comparing the predictive performance of these fetal growth charts have focused primarily on fetal growth restriction and, consequently, small‐for‐gestational‐age (SGA) infants at birth^15^, ^16^, ^17^. A previous study assessed the predictive performance of these charts for LGA infants at birth but did not focus on pregnant individuals with pregestational diabetes^18^. Regardless of the growth chart used, sonographically estimated fetal weight (EFW) can deviate from actual BW, and more so with LGA infants^19^, ^20^, ^21^. Inclusion of clinical factors may improve the predictive performance of growth charts^22^, ^23^, ^24^. For example, poor glycemic control, as assessed by hemoglobin A1c (HbA1c), is associated with LGA infants in pregnancies with pregestational diabetes^25^, ^26^, ^27^, ^28^; however, whether the combination of HbA1c measurement with growth charts results in improved prediction of LGA remains to be studied.

The objectives of the current study were to compare the ability of three fetal growth charts (Hadlock, NICHD race/ethnicity‐specific and FMF) to predict LGA at birth in pregnant individuals with pregestational diabetes, and to determine whether their predictive performance improved with the addition of HbA1c.

## METHODS

### Study setting and participants

We conducted a retrospective cohort study of pregnant individuals presenting with pregestational diabetes between January 2012 and December 2016 at a tertiary care academic medical center in the midwestern USA (The Ohio State University Wexner Medical Center, Columbus, OH, USA). Pregestational diabetes was confirmed if there was a prior diagnosis before pregnancy of either Type‐1 or Type‐2 diabetes, based on International Classification of Diseases, ninth revision (ICD‐9) codes^29^, which was then confirmed by review of the electronic health record. Pregnant individuals without a formal diagnosis of pregestational diabetes but with an elevated HbA1c ≥ 6.5% within 12 weeks before or after the last menstrual period were categorized as having Type‐2 diabetes (*n* = 6)^30^. We excluded individuals with early‐onset gestational diabetes (*n* = 7), which was defined as a 1‐h, 50‐g glucose challenge test ≥ 200 mg/dL at < 20 weeks' gestation, or two elevated values on a 3‐h, 100‐g glucose‐tolerance test at < 20 weeks' gestation^31^. The current analysis was restricted further to singleton pregnancies with a non‐anomalous live birth that underwent prenatal growth ultrasound between 28 + 0 and 36 + 6 weeks of gestation. Pregnancies that delivered outside our hospital were excluded as it was not possible to ascertain their outcome data. This study was approved by the institutional review board of the Ohio State University, Columbus, OH, USA (study ID#: 2017H0145; approval date: 4/27/2017). Informed patient consent was not required due to retrospective chart abstraction and all patient data were deidentified. This study was performed in accordance with the Standards for Reporting Diagnostic Accuracy Studies (STARD) guidelines^32^.

Briefly, pregnant individuals were identified through an electronic data query. Three study authors (M.K.K., M.M.F., C.A.W.) retrieved manually demographic and clinical data from the electronic health record of each patient into a secure electronic database. Gestational age was established based on current guidelines^33^, using the best obstetric estimate. The following covariates were collected: age at delivery, body mass index (BMI) at delivery, self‐reported race/ethnicity, parity, chronic hypertension, White's classification of diabetes in pregnancy^34^ and diabetes type. In addition, obstetric outcomes, including mode of delivery, BW and adverse outcomes, including pre‐eclampsia, neonatal shoulder dystocia and neonatal hypoglycemia < 35 mg/dL, were retrieved from the maternal and child delivery discharge summaries.

### Exposure

The primary exposure was suspected LGA, defined as an EFW ≥ 90^th^ percentile on prenatal ultrasound, calculated using the Hadlock, NICHD race/ethnicity‐specific and FMF growth charts. The Hadlock growth chart was selected because it is used widely in current clinical practice in the USA and has been shown to be accurate in comparison to older growth charts^35^, ^36^. For the current study, we utilized Hadlock Formula C (Hadlock C: log_10_ BW = 1.335 − 0.0034 × abdominal circumference (AC) × femur length (FL) + 0.0316 × biparietal diameter + 0.0457 × AC + 0.1623 × FL), which has demonstrated the best performance among the Hadlock formulas^10^, ^37^. The NICHD growth chart was selected because of its focus on including a racially and ethnically diverse contemporary population^11^, and because prior research has suggested it has a higher screen‐positive rate for EFW ≥ 90^th^ percentile compared with other growth charts^17^. The FMF growth chart was selected because it may improve the ability to predict LGA compared with the Hadlock growth chart in low‐risk individuals without pregestational diabetes^18^. At the time at which the current study was conducted, our institution utilized the Brenner growth chart, and has more recently transitioned to the Hadlock growth chart.

We then assessed whether the ability to predict LGA by a given fetal growth chart could be improved further by inclusion of HbA1c as a continuous measure in the second half of pregnancy (i.e. > 20 weeks of gestation). As part of our diabetes‐in‐pregnancy program, providers were encouraged to check HbA1c at least once per trimester. While threshold values of HbA1c associated with adverse pregnancy outcomes have been proposed^38^, given a linear association between HbA1c and LGA and the potential loss of information with a dichotomized variable, we assessed primarily HbA1c as a continuous percentage^25^. In addition, to enhance clinical utility, HbA1c was assessed secondarily as a dichotomous variable as < 6.5% *vs* ≥ 6.5%. HbA1c cut‐off values of 6.0% and 6.5% have been proposed by professional society guidelines^38^, and a cut‐off of 6.5% is consistent with prior studies in this clinical cohort^39^, ^40^. For cases in which more than one HbA1c measurement was available between 20 weeks and delivery, the measurement closest to the time of delivery was used. A standard assay was used for HbA1c (Variant II Turbo HbA1c Kit (normal range, 4.7–5.6%; coefficient of variation, < 3%); Bio‐Rad Laboratories, Hercules, CA, USA)^41^.

### Outcome

The primary outcome was LGA at birth, defined as a BW in g ≥ 90^th^ percentile using the updated infant sex‐specific 2017 USA natality reference^42^. This newer reference chart included the most recent sociodemographic composition in the USA and also addressed concerns regarding the validity of prior last‐menstrual‐period‐based references by using an obstetric estimate‐based reference.

### Statistical analysis

The performance of the three growth charts to predict LGA at birth was assessed using the area under the receiver‐operating‐characteristics (ROC) curve (AUC). Sensitivity, specificity, positive predictive value (PPV) and negative predictive value (NPV) were also estimated. The AUC, which ranges from 0 to 1, is a measure of the predictive performance of a test^43^. Differences between the AUCs obtained from each growth chart were compared using the DeLong method^44^. In the subset of participants with available HbA1c data (306/358), we assessed whether the performance of each growth chart could be improved with inclusion of HbA1c and compared the differences between the AUCs (growth chart alone *vs* growth chart with HbA1c). Predicted probabilities, derived from a logistic regression model, were used to construct ROC curves and to compute the AUCs.

In sensitivity analyses, because fetal biometry is less accurate in the setting of maternal obesity^45^ and when performed more remote from delivery (i.e. in the early third trimester)^46^, we performed again the above analyses restricted to individuals with obesity (BMI ≥ 30 kg/m^2^ at delivery) (*n* = 247) and in those with ultrasound assessment performed after the mid third trimester (> 32 weeks' gestation) (*n* = 188). Because HbA1c data were available in only a subset of participants (*n* = 306), we reperformed the analysis comparing the growth charts alone without HbA1c restricted to this subset due to possible selection bias when restricted to those with HbA1c data. Statistical analysis was performed using STATA version 16.1 (Stata Corp., College Station, TX, USA).

## RESULTS

**Figure 1:**
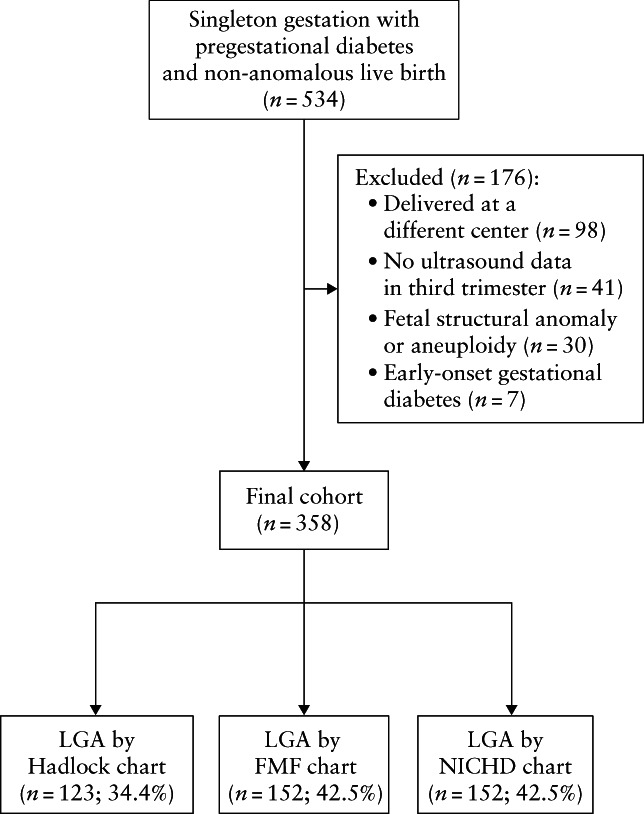
Flowchart showing inclusion in study of pregnant individuals with pregestational diabetes who received diabetes care during pregnancy at Ohio State University Wexner Medical Center, Columbus, OH, USA and proportion predicted prenatally to have large‐for‐gestational‐age (LGA) infant at birth based on the Hadlock, Fetal Medicine Foundation (FMF) and National Institutes of Child Health and Human Development (NICHD) growth charts.

During the study period, 534 individuals with pregestational diabetes and a singleton gestation received diabetes care in pregnancy at our center. Those who delivered at a different medical center (*n* = 98), did not undergo an ultrasound assessment in the third trimester (*n* = 41), or had a fetal structural or chromosomal anomaly (*n* = 30) or early‐onset gestational diabetes (*n* = 7) were excluded (Figure 1). Thus, the final sample comprised 358 individuals, of whom 34% had Type‐1 and 66% Type‐2 diabetes. HbA1c data were available in 306/358 individuals; the 52 individuals lacking HbA1c data did not differ in age, self‐reported race and ethnicity, parity, BMI or type of diabetes from those included (all *P* > 0.05).

### Participant characteristics

The mean ± SD maternal age was 31.7 ± 6.3 years and the mean BMI at delivery was 35.4 ± 9.5 kg/m^2^. Overall, 189/357 (53%) individuals were non‐Hispanic white and 132/358 (37%) were nulliparous (Table 1). Most participants (208/355; 59%) were categorized as class B according to White's classification of diabetes in pregnancy, and 246/305 (81%) were on an insulin‐containing diabetes treatment regime at delivery. The mean HbA1c was 6.4% ± 1.2% and 122/306 (40%) individuals had a HbA1C ≥ 6.5%, which was assessed at a mean gestational age of 31.1 ± 3.8 weeks. At the time of delivery, 242/357 (68%) individuals had a Cesarean delivery, which was indicated due to fetal macrosomia in 19/242 (8%) cases, 94/358 (26%) participants were diagnosed with pre‐eclampsia, and 21/115 (18%) of vaginal deliveries were complicated by shoulder dystocia. One‐fifth (74/358; 21%) of infants had a BW of ≥ 4000 g and 116/358 (32%) were diagnosed with neonatal hypoglycemia.

**Table 1:** Demographic and clinical characteristics in total cohort of pregnant individuals with pregestational diabetes and in those suspected to have large‐for‐gestational‐age infant at birth by the Hadlock, Fetal Medicine Foundation (FMF) and National Institutes of Child Health and Human Development (NICHD) growth charts

Characteristic	Overall (*n* = 358)	Fetal growth chart		
		Hadlock *(n* = 123)	FMF (*n* = 152)	NICHD *(n* = 152)
Age (years)	31.7 ± 6.3	31.0 ± 6.6	31.0 ± 6.4	31.3 ± 6.5
Nulliparous	132 (36.9)	44 (35.8)	60 (39.5)	49 (32.2)
Race and ethnicity				
Non‐Hispanic white	189/357 (52.9)	75 (61.0)	93 (61.2)	76 (50.0)
Non‐Hispanic black	100/357 (28.0)	29 (23.6)	37 (24.3)	47 (30.9)
Hispanic/Latina	21/357 (5.9)	9 (7.3)	9 (5.9)	10 (6.6)
Non‐Hispanic Asian/Pacific Islander	47/357 (13.2)	10 (8.1)	13 (8.6)	19 (12.5)
BMI at delivery (kg/m^2^) (*n* = 357)	35.4 ± 9.5	36.1 ± 9.5	35.5 ± 9.3	35.9 ± 9.2
Normal or overweight	111 (31.0)	38 (30.9)	47 (30.9)	45 (29.6)
Class‐I obesity* or greater	247 (69.0)	85 (69.1)	105 (69.1)	107 (70.4)
Pregestational diabetes				
Type 1	120 (33.6)	43 (35.0)	61 (40.1)	52 (34.2)
Type 2	237 (66.4)	80 (65.0)	91 (59.9)	100 (65.8)
Gestational age at delivery (weeks)	37.2 ± 2.0	37.9 ± 1.7	37.0 ± 1.7	37.1 ± 1.7
White's diabetes classification†				
B	208/355 (58.6)	72/121 (59.5)	84/150 (56.0)	91/150 (60.7)
C	75/355 (21.1)	31/121 (25.6)	41/150 (27.3)	37/150 (24.7)
D	37/355 (10.4)	10/121 (8.3)	14/150 (9.3)	11/150 (7.3)
R/F/RF	35/355 (9.9)	8/121 (6.6)	11/150 (7.3)	11/150 (7.3)
Diabetes pharmacotherapy at delivery				
Insulin	237/305 (77.7)	97 (78.9)	125 (82.2)	120 (78.9)
Insulin and oral agents‡	9/305 (3.0)	3 (2.4)	3 (2.0)	3 (2.0)
Oral agents only‡	43/305 (14.1)	16 (13.0)	17 (11.2)	21 (13.8)
None	16/305 (5.2)	7 (5.7)	7 (4.6)	8 (5.3)
GA at ultrasound assessment (weeks)	31.6 ± 1.5	31.4 ± 1.2	31.5 ± 1.3	31.4 ± 1.3
GA at HbA1c assessment (weeks)§	31.1 ± 3.8	31.2 ± 3.6	31.1 ± 3.6	31.3 ± 3.7
HbA1c (%)§	6.4 ± 1.2	6.5 ± 1.2	6.5 ± 1.2	6.5 ± 1.2
HbA1c ≥ 6.5%	122/306 (39.9)	50/97 (51.5)	63/132 (47.7)	59/131 (45.0)
Adverse pregnancy outcome				
Birth weight ≥ 4000 g	74 (20.7)	56 (45.5)	63 (41.4)	58 (38.2)
Neonatal hypoglycemia < 35 mg/dL	116 (32.4)	53 (43.1)	65 (42.8)	57 (37.5)
Neonatal shoulder dystocia among VDs	21/115 (18.3)	10/30 (33.3)	12/37 (32.4)	14/45 (31.1)
CS	242/357 (67.8)	93 (75.6)	115 (75.7)	107 (70.4)
CS for macrosomia	19/242 (7.9)	13/93 (14.0)	15/115 (13.0)	13/107 (12.1)
Pre‐eclampsia	94 (26.3)	32 (26.0)	42 (27.6)	42 (27.6)

Data are given as mean ± SD, *n* (%) or *n*/*N* (%).

* Defined as body mass index (BMI) > 30 kg/m^2^.

† White's classification of diabetes in pregnancy: B, diabetes duration < 10 years; C, diabetes duration 10–19 years; D, diabetes duration ≥ 20 years; R, diabetes with retinopathy; F, diabetes with nephropathy; RF, diabetes with retinopathy and nephropathy.

‡ Oral agents include metformin or glyburide.

§ Data available in 306 participants. CS, Cesarean section; GA, gestational age; HbA1c, glycosylated hemoglobin; VD, vaginal delivery.

A total of 147 (41%) infants were diagnosed with LGA at birth. The mean gestational age at ultrasound assessment was 31.6 ± 1.5 weeks. By prenatal ultrasound, LGA was suspected in 152 (42.5%) pregnancies by the FMF growth chart, 152 (42.5%) by the NICHD growth chart, and 123 (34.4%) by the Hadlock growth chart (Table 1).

### Performance of FMF, NICHD and Hadlock growth charts to identify LGA

The FMF growth chart had the highest sensitivity (77% *vs* 69% (NICHD) *vs* 63% (Hadlock)), whereas the Hadlock growth chart had the highest specificity (86% *vs* 76% (NICHD) *vs* 82% (FMF)) for predicting LGA at birth (Table 2). The FMF and Hadlock growth charts had a higher PPV compared with the NICHD growth chart (74% (FMF) and 76% (Hadlock) *vs* 67% (NICHD)). The FMF growth chart had the highest NPV of 84% compared with 77% for the Hadlock and 78% for the NICHD charts.

**Table 2:** Performance of estimated fetal weight ≥ 90^th^ percentile derived from Fetal Medicine Foundation (FMF), National Institutes of Child Health and Human Development (NICHD) and Hadlock fetal growth charts for prediction of large‐for‐gestational‐age infant at birth in 358 pregnant individuals with pregestational diabetes

Fetal growth chart	Sensitivity (%)	Specificity (%)	PPV (%)	NPV (%)	AUC	*P* *
FMF	76.9 (69.2–83.4)	81.5 (75.6–86.5)	74.3 (66.6–81.1)	83.5 (77.7–88.3)	0.79 (0.74–0.84)	< 0.001†; < 0.01‡
NICHD	68.7 (60.5–81.4)	75.8 (69.5–81.4)	66.5 (58.3–73.9)	77.7 (71.4–83.2)	0.72 (0.68–0.77)	0.10‡
Hadlock	63.2 (54.6–71.1)	85.8 (80.3–90.2)	75.6 (67.0–82.9)	77.0 (71.1–82.2)	0.75 (0.70–0.79)	—

Values in parentheses are 95% CI.

* Receiver‐operating‐characteristics (ROC) curves were compared using the DeLong method^44^.

† *vs* NICHD.

‡ *vs* Hadlock. AUC, area under the ROC curve; NPV, negative predictive value; PPV, positive predictive value.

**Figure 2:**
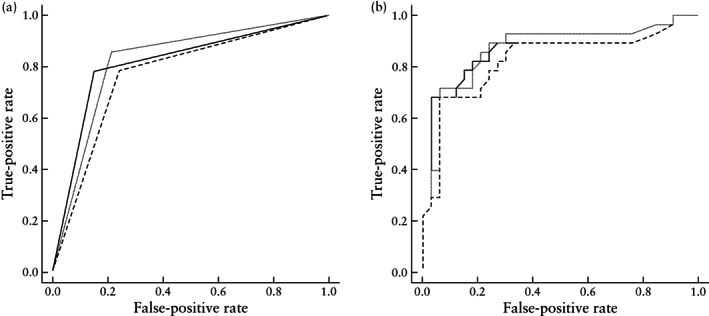
Receiver‐operating‐characteristics curves for prediction of large‐for‐gestational‐age infant at birth in pregnant individuals with pregestational diabetes, using estimated fetal weight ≥ 90^th^ percentile derived from the Fetal Medicine Foundation (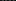), National Institutes of Child Health and Human Development (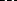) and Hadlock (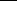) growth charts, alone (a) and in combination with hemoglobin A1c as a continuous measure (b).

The FMF growth chart was statistically more predictive of LGA at birth (AUC, 0.79 (95% CI, 0.74–0.84)) compared with the NICHD (AUC, 0.72 (95% CI, 0.68–0.77); *P* < 0.001) and Hadlock (AUC, 0.75 (95% CI, 0.70– 0.79); *P* < 0.01) growth charts (Table 2, Figure 2a). The AUCs of the Hadlock and NICHD growth charts for predicting LGA at birth were similar.

### Performance of FMF, NICHD and Hadlock growth charts with HbA1c to identify LGA

The FMF growth chart combined with HbA1c had the highest sensitivity (79% *vs* 68% (NICHD) *vs* 64% (Hadlock)), and the Hadlock growth chart with HbA1c had the highest specificity (83% *vs* 76% (NICHD) *vs* 80% (FMF)) for predicting LGA at birth (Table 3). When including HbA1c, the FMF and Hadlock growth charts had a higher PPV compared with the NICHD growth chart (73% (FMF) and 73% (Hadlock) *vs* 66% (NICHD)), and the FMF growth chart had the highest NPV (84% *vs* 77% (Hadlock) and 77% (NICHD)).

**Table 3:** Performance of Fetal Medicine Foundation (FMF), National Institutes of Child Health and Human Development (NICHD) and Hadlock fetal growth charts in combination with hemoglobin A1c (HbA1c) for prediction of large‐for‐gestational‐age infant at birth in 306 pregnant individuals with pregestational diabetes and HbA1c data available

Fetal growth chart	Sensitivity (%)	Specificity (%)	PPV (%)	NPV (%)	AUC	*P* *
HbA1c as a continuous variable						
FMF	78.6 (70.4–85.4)	80.0 (73.4–85.6)	73.3 (65.0–80.6)	84.2 (77.9–89.3)	0.85 (0.81–0.90)	< 0.001†; 0.02‡
NICHD	67.5 (58.5–75.5)	76.1 (69.2–82.1)	66.4 (57.5–74.5)	77.0 (70.1–82.9)	0.79 (0.73–0.84)	0.06‡
Hadlock	63.5 (54.4–71.9)	83.3 (77.1–88.5)	72.7 (63.4–80.8)	76.5 (70.0–82.3)	0.81 (0.76–0.86)	—
HbA1c ≥ 6.5%						
FMF	77.0 (68.6–84.0)	80.6 (74.0–86.1)	73.5 (65.1–80.8)	83.3 (76.9–88.5)	0.84 (0.79–0.88)	< 0.001†; 0.01‡
NICHD	68.3 (59.4–76.3)	75.0 (68.0–81.1)	65.7 (56.9–73.7)	77.1 (70.2–83.1)	0.78 (0.73–0.83)	0.13‡
Hadlock	61.9 (52.8–70.4)	85.6 (79.6–90.3)	75.0 (65.6–83.0)	76.2 (69.8–81.9)	0.80 (0.75–0.85)	—

Values in parentheses are 95% CI.

* Receiver‐operating‐characteristics (ROC) curves were compared using the DeLong method^44^.

† *vs* NICHD.

‡ *vs* Hadlock. AUC, area under the ROC curve; NPV, negative predictive value; PPV, positive predictive value.

After inclusion of HbA1c as a continuous variable, the FMF growth chart (AUC, 0.85 (95% CI, 0.81– 0.90)) was statistically more predictive of LGA at birth than the NICHD (AUC, 0.79 (95% CI, 0.73–0.84); *P* < 0.001) and Hadlock (AUC, 0.81 (95% CI, 0.76–0.86); *P* = 0.02) growth charts (Table 3, Figure 2b). There was no statistical difference between the accuracy of the Hadlock and NICHD growth charts (*P* = 0.06). These results held when using a HbA1c threshold of ≥ 6.5% (Table 3).

The predictive performance of all three growth charts improved significantly with the inclusion of HbA1c compared with each growth chart alone (*P* < 0.001 for all).

### Sensitivity analysis

The primary results comparing the three growth charts alone held when restricting the analysis to individuals with obesity (AUC > 0.7) and those with ultrasound assessment ≥ 32 weeks' gestation (AUC > 0.7) (Table 4). The primary results also held when restricted to the subset of individuals with HbA1c data due to concern for selection bias. In all three sensitivity analyses, the FMF was more predictive of LGA at birth in comparison to the Hadlock and NICHD growth charts.

**Table 4:** Predictive performance of Fetal Medicine Foundation (FMF), National Institutes of Child Health and Human Development (NICHD) and Hadlock fetal growth charts for large‐for‐gestational‐age infant at birth in pregnant individuals with pregestational diabetes, in those with body mass index (BMI) at delivery ≥ 30 kg/m^2^ (*n* = 247), those with ultrasound assessment performed ≥ 32 weeks (*n* = 188) and those with hemoglobin A1c (HbA1c) data available (*n* = 306)

Fetal growth chart	AUC (95% CI)	*P* *
BMI at delivery ≥ 30 kg/m^2^		
FMF	0.76 (0.71–0.82)	< 0.01†
		0.02‡
NICHD	0.71 (0.65–0.77)	0.46‡
Hadlock	0.72 (0.66–0.78)	—
US performed ≥ 32 weeks		
FMF	0.82 (0.76–0.87)	< 0.001†
		< 0.001‡
NICHD	0.73 (0.66–0.79)	0.80‡
Hadlock	0.72 (0.66–0.78)	—
Patients with HbA1c data		
FMF	0.79 (0.74–0.83)	< 0.001†
		< 0.01‡
NICHD	0.72 (0.66–0.77)	0.18‡
Hadlock	0.74 (0.69–0.79)	—

* Receiver‐operating‐characteristics (ROC) curves were compared using the DeLong method^44^.

† *vs* NICHD.

‡ *vs* Hadlock. AUC, area under the ROC curve; US, ultrasound assessment.

## DISCUSSION

### Study findings

Among pregnant individuals with pregestational diabetes, all three assessed fetal growth charts (FMF, NICHD and Hadlock) demonstrated good predictive ability to identify LGA infants at birth (AUCs > 0.7), with the FMF growth chart having the highest AUC. The inclusion of HbA1c improved further the predictive performance of all three growth charts (AUCs ≥ 0.8).

### Previous research

The findings of the current study in a population with pregestational diabetes, most of whom had obesity, and with a high frequency of LGA (> 40%), are consistent with prior studies that compared the performance of fetal growth charts in lower‐risk pregnant individuals, including those without diabetes and consequently with a lower risk of LGA (12%)^18^. In their low‐risk pregnant population, Duncan *et al*.^18^ found that the FMF growth chart had the highest accuracy for detection of LGA (AUC, 0.8). Similarly, in an obese population without diabetes, Verger *et al*.^23^ demonstrated adequate sensitivity (85%) and specificity (72%) of the FMF growth chart to identify LGA infants.

Furthermore, the current study found that inclusion of HbA1c, either as a continuous measure or based on a cut‐off value of 6.5%, improved the prediction of LGA with all three assessed growth charts. HbA1c is a marker of glycemic control in pregnancy and maternal hyperglycemia results in changes to fetal anthropometric measurements^47^. HbA1c is associated with an increased risk of LGA infants in pregnant individuals with pregestational diabetes^25^. In current clinical practice, HbA1c is used frequently to titrate pharmacotherapy and assess treatment response for diabetes in pregnancy^30^. These results suggest that HbA1c may also be used as a marker to assess the risk of fetal overgrowth in combination with prenatal ultrasound.

### Clinical implications

Providing an accurate prenatal diagnosis of suspected LGA in the setting of pregestational diabetes can help determine the risk of adverse perinatal outcome and assist with patient counseling^48^. The FMF growth chart had the best performance for predicting LGA at birth in our population. Prior studies conducted in low‐risk populations suggest that the FMF growth chart has a higher false‐positive rate, may result in increased obstetric interventions at delivery and a higher frequency of SGA, which is a known complication of pregestational diabetes^17^, ^18^. Whether improved prenatal prediction of LGA results in reduced adverse maternal and neonatal outcomes associated with dysglycemia, including Cesarean delivery, shoulder dystocia and neonatal hypoglycemia, will require further study. With regard to patient counseling, if shown to be accurate, such models may be able to better identify pregnancies in need of Cesarean delivery to prevent shoulder dystocia in this population with a high rate of Cesarean delivery^2^, ^48^.

Moreover, the findings of the current study may inform the development of future predictive models aimed at improving the diagnosis of LGA. The combination of HbA1c with fetal biometry may improve the ability to detect pregnancies at the highest risk of LGA, and hence, potentially mitigate some of the consequences of fetal overgrowth with pharmacotherapy titration, which will require further study.

### Limitations

First, ultrasound assessments performed more distant from delivery are more likely to be inaccurate for estimating LGA at birth. We utilized ultrasound data in the third trimester (mean of 32 weeks' gestation) and when more than one ultrasound examination was available, we utilized the one closest to delivery. Fetal biometry with ultrasound, even when conducted proximate to delivery, can be an inaccurate measure of BW, particularly for suspected LGA^21^, ^49^. The error rate also increases in the setting of obesity^45^. The primary analysis held in sensitivity analyses restricted to participants with obesity and those with ultrasound assessment performed ≥ 32 weeks' gestation. These results should be replicated in datasets with ultrasound data obtained closer to delivery to assess whether more accurate prediction is possible with macrosomia (i.e. BW > 4000 g or > 4500 g). Second, we did not evaluate the interoperator variability among sonographers in this retrospective study, although all sonographers were certified by the American Registry for Diagnostic Medical Sonography^50^. Third, we assessed HbA1c during a relatively wide interval during the second half of pregnancy. The optimal interval for HbA1c assessment in pregnancy, particularly with regard to its association with LGA, remains to be defined. Fourth, selection bias is possible as HbA1c data were not available for all participants. However, the primary analysis comparing the three growth charts alone held in the subsample with HbA1c data, and the sociodemographic and clinical characteristics of participants without HbA1c data did not differ significantly from those of individuals with such data available. Fifth, this study did not assess the relative difference in the association between suspected LGA by each growth chart and adverse outcome. However, the associations between LGA, dysglycemia and adverse pregnancy outcome are well characterized in pregnancies with pregestational diabetes. Finally, this was a single institution study in which participants received both pregnancy and diabetes care together, and these results will need to be replicated in other practice settings.

### Conclusions

Among pregnant individuals with pregestational diabetes, all three assessed fetal growth charts (FMF, NICHD and Hadlock) demonstrated good predictive ability for identifying LGA infants at birth. The FMF growth chart had the best predictive performance for LGA at birth in comparison to the NICHD and Hadlock growth charts. In addition, inclusion of HbA1c improved the predictive performance for all three growth charts.

## Acknowledgment

K.K.V. was supported by the Care Innovation and Community Improvement Program at The Ohio State University, Columbus, OH, USA.

## Data Availability

Data are available from the corresponding author upon reasonable request.
